# American Association of Obstetricians and Gynecologists

**Published:** 1897-09

**Authors:** 


**A Monthly Review of Medicine and Surgery.**

*EDITORS:*

THOMAS LOTHROP, M. D. - - WM. WARREN POTTER, M. D.

All communications, whether of a literary or business nature, should be addressed to the managing editor: 284 FRANKLIN STREET, BUFFALO, N. Y.

VOL. XXXVII. SEPTEMBER, 1897. No. 2.

### AMERICAN ASSOCIATION OF OBSTETRICIANS AND GYNECOLOGISTS.

IT HAS been thought by many experienced in such matters to be impracticable to hold a successful meeting of a special medical society in the summer season at a pleasure resort. This association, however, has demonstrated the possibility of doing so. It held its tenth annual session at the Cataract House, Niagara Falls, August 17–20, 1897, and it turned out to be one of the most successful and interesting meetings it ever held. Not only was there a large attendance of the Fellows, but the medical profession, both from immediate and remote points, attended in generous force to listen to a large group of interesting papers, followed in most instances by equally interesting discussions.

One of the principal elements of successful work is suitable weather, and at Niagara, during the period mentioned, the temperature was cool, the air inspiriting to mental effort, and the whole environment bordered on the ideal. The papers, as usual in this association, dealt forcfully with a wide range of subjects in obstetrics, gynecology, abdominal and pelvic surgery. Many of the discussions were exhaustive in character, spirited in manner and instructive in teaching. It would be difficult to distinguish or differentiate those that best answer this description when all were more or less filled with animation and research. When the work done at this meeting is published those who read it will be able to judge best for themselves of its character and usefulness.

Turning now to the social part of the meeting it is proper to remark that the Niagara Falls Academy of Medicine contributed largely to the enjoyment of the occasion, and especially to the entertainment of the visiting ladies. As soon as it was announced last year that the tenth annual meeting would be held at Niagara Falls, the Academy of Medicine, individually and collectively, tendered its services, to be used in any manner available to promote the interests of the association and render its visit a pleasant one. It held numerous meetings and appointed various committees to further these ends.

Dr. W. R. Campbell, president of the Academy, delivered the address of welcome on the morning of the first day, while a committee was in constant attendance on the meetings to furnish information to the visitors and to assist them in any manner in which their services could be made available. A tally-ho coach left the Cataract House every morning at ten o'clock, loaded with visiting ladies and escorted by members of the Academy of Medicine. This afforded a pleasant opportunity to see the Falls from various view points, and was much appreciated by the ladies. On Wednesday afternoon, the second day of the meeting, the Niagara Electric Power company, by a special invitation, afforded the visitors an opportunity of inspecting its wonderful plant. At the instance of the Academy of Medicine the Gorge and the Canadian electric railways made a special round-trip rate, giving all a view of the river on both sides, above and below the Falls. There is no excursion to be taken within the limits of time and distance that affords a finer view of nature in heroic grandeur than this one, all of which apparently was appreciated to the utmost by the Association and its guests.

The annual dinner was given at the Cataract House, Thursday evening at 8 o'clock. The president, Dr. James F. W. Ross, presided and introduced the several speakers in appropriate terms. The following addresses were made: "The American Association of Obstetricians and Gynecologists," Dr. James F. W. Ross, Toronto; "The State of New York and the Niagara Falls Reservation," Hon. Thomas V. Welch, Niagara Falls; "The Niagara Falls Academy of Medicine," Dr. Charles G. Stivers, Niagara Falls; "The Niagara Frontier in History," Peter A. Porter, Esq., Niagara Falls; "The State and the Medical Profession," Hon. Rowland B. Mahany, Buffalo; "The American Medical Association," Dr. Joseph M. Mathews, Louisville. The eloquence of the speakers was frequently interrupted with outbursts of applause, and it is pertinent to add that seldom has post prandial oratory interested an audience from beginning to end in a greater degree.

Instrumental music was furnished by the Cataract House orchestra and the speaking was interspersed with vocal selections by Mr. John Broughton, bass soloist, and the Niagara Falls quartette. The musical features of the occasion were especially fine and were particularly enjoyed by the diners.

The election of officers for the ensuing year resulted as follows : president, Dr. Charles A. L. Reed, of Cincinnati ; vice-presidents, Dr. Richard Douglas, of Nashville, and Dr. Walter B. Dorsett, of St. Louis ; secretary, Dr. William Warren Potter, of Buffalo ; treasurer, Dr. Xavier Oswald Werder, of Pittsburg ; executive council, Drs. Lewis Samuel McMurtry, of Louisville, Albert Vander Veer, Albany, J. Henry Carstens, Detroit, William E. B. Davis, Birmingham, and John Milton Duff, Pittsburg.

The next meeting will be held at Pittsburg, September 20, 21 and 22, 1898. The association adjourned at 1 o'clock P. M. on Friday, after one of the most interesting meetings in its history. The management of the Cataract House most courteously contributed its full share toward this result.

